# Reference gene selection for gene expression studies using RT-qPCR in virus-infected planthoppers

**DOI:** 10.1186/1743-422X-8-308

**Published:** 2011-06-16

**Authors:** Guillermo A Maroniche, Mónica Sagadín, Vanesa C Mongelli, Graciela A Truol, Mariana del Vas

**Affiliations:** 1 Instituto de Biotecnología, CICVyA, Instituto Nacional de Tecnología Agropecuaria (IB-INTA), Las Cabañas y Los Reseros s/n, Hurlingham CP 1686, Buenos Aires, Argentina; 2 Instituto de Fitopatología y Fisiología Vegetal, Instituto Nacional de Tecnología Agropecuaria (IFFIVE-INTA), Camino 60 cuadras km 5 1/2, X5020ICA, Córdoba, Argentina

## Abstract

**Background:**

Planthoppers not only severely affect crops by causing mechanical damage when feeding but are also vectors of several plant virus species. The analysis of gene expression in persistently infected planthoppers might unveil the molecular basis of viral transmission. Quantitative real-time RT-PCR (RT-qPCR) is currently the most accurate and sensitive method used for quantitative gene expression analysis. In order to normalize the resulting quantitative data, reference genes with constant expression during the experimental procedures are needed.

**Results:**

Partial sequences of the commonly used reference genes actin (ACT), α1-tubulin (TUB), glyceraldehyde 3-phosphate dehydrogenase (GAPDH), elongation factor 1 alpha (EF1A), ribosomal protein S18 (RPS18) and polyubiquitin C (UBI) from *Delphacodes kuscheli*, a planthopper capable of persistently transmitting the plant fijivirus *Mal de Río Cuarto virus *(MRCV), were isolated for the first time. Specific RT-qPCR primers were designed and the expression stability of these genes was assayed in MRCV-infective and naïve planthoppers using geNorm, Normfinder and BestKeeper tools. The overall analysis showed that UBI, followed by 18S and ACT, are the most suitable genes as internal controls for quantitative gene expression studies in MRCV-infective planthoppers, while TUB and EF1A are the most variable ones. Moreover, EF1A was upregulated by MRCV infection.

**Conclusions:**

A RT-qPCR platform for gene expression analysis in the MRCV-infected planthopper vector *Delphacodes kuscheli *was developed. Our work is the first report on reference gene selection in virus-infected insects, and might serve as a precedent for future gene expression studies on MRCV and other virus-planthopper pathosystems.

## Background

Insect genomics is a relatively new field of research. Since the report of the fruit fly genome sequence in 2000 [1], several other insect genomes have been completed including three mosquitoes species, silkworm, honeybee, red flour beetle, pea aphid, three closely related parasitoid wasps and human body louse [2-5]. A considerable number of other insect genomes are in progress [6]. In parallel, high throughput tools for functional studies in insects are being developed. For example, Gateway-based vectors applicable for the subcellular localization analysis of proteins in cultured *Bombyx mori *cells became available [7]. Also important, new insect promoters for gene expression studies are being isolated and characterized [8] and an insect two-hybrid system to analyze protein interactions in cultured insect cells has been reported [9]. Planthoppers (order *Hemiptera*, superfamily *Fulgoroidea*) are severe pests of plants because of their sucking damage and ability to transmit at least 18 phytopathogenic viruses [10]. For example, the brown planthopper *Nilaparvata lugens *is a serious pest of rice in tropical Asia and propagatively transmits *Rice ragged stunt virus *(RRSV, *Oryzavirus *genus of the *Reoviridae *family), *Rice grassy stunt virus *(RGSV, genus *Tenuivirus*) [11] and *Nilaparvata lugens reovirus *(NLRV, *Fijivirus *genus of the *Reoviridae *family) [12]. In turn, the small brown planthopper *Laodelphax striatellus *transmits *Rice stripe virus *(RSV, genus *Tenuivirus*) and *Rice black streak dwarf virus *(RBSDV, *Fijivirus *genus of the *Reoviridae *family) [13,14] and the planthopper *Peregrinus maidis *transmits *Maize mosaic virus *(MMV: *Nucleorhabdovirus *genus of the *Rhabdoviridae *family) [15]. Another planthopper species, *Delphacodes kuscheli*, is a natural vector that propagatively transmits *Mal de Río Cuarto virus *(MRCV, *Fijivirus *genus of the *Reoviridae *family). MRCV causes the most important maize disease in Argentina and its genome sequence has recently been completed [16-19].

Although no planthopper genonome sequencing project has yet been announced, great progress has been made with the sequencing of more than 37,000 *Nilaparvata lugens *ESTs originated from various tissues [20] and of 85,526 unigenes from different developmental stages, sexes and wing forms using short-read sequencing technology combined with a tag-based digital gene expression system [21]. This information will certainly contribute to the unveiling of molecular events underlying virus transmission by insects. In particular, very little is known regarding the effects of persistent virus infection on planthoppers transcriptome and new robust and accurate methods for gene expression studies will be required. Most recently, massive *Laodelphax striatellus *parallel pyrosequencing-based transcriptome analyses was performed in both naïve and RSV-infected insects and several planthopper genes diferentially expressed during virus infection were identified [22].

The relative quantification of mRNA levels by RT-qPCR is currently an extensively used technique, in which reliable quantification depends on the use of one or more stably expressed endogenous genes, usually housekeeping genes, as internal controls. However, because housekeeping gene expression is not always stable and might vary depending on the samples or treatments, it is necessary to first study the stability of several endogenous gene expression in order to select suitable internal references [23]. Thus, stability analysis of reference genes in diverse conditions and organisms are rapidly increasing [24-34]. Even though the behavior of insect housekeeping genes has been studied in bacterially challenged bees [30] and in *Tribolium *beetles infected with fungus [28], this kind of information is lacking for virus-infected insects. In addition, there is no information currently available on reference gene stability in *Delphacodes kuscheli *or any other planthopper for accurate quantification of mRNA levels by RT-qPCR. Our study aimed to examine the stability of potential reference genes expression upon MRCV infection of single *D. kuscheli *planthoppers and to rank them according to their reliability as internal controls. As a result, a robust RT-qPCR platform was set up for analyzing changes on gene expression upon virus infection in individual planthoppers.

## Methods

### Source of virus and one-to-one transmission assays

The RC MRCV isolate from the endemic disease area (County of Río Cuarto, Province of Córdoba, Argentina) was maintained on oat (*Avena sativa *L.) plants through successive transmissions by *D. kuscheli *as described previously [35]. To obtain MRCV-infective insects, 5 III instar *D. kuscheli *nymphs were fed on a MRCV-infected oat plant for two days. Next, the insects were moved to a cage and fed on uninfected wheat (*Triticum aestivum *cv Prointa federal) seedlings (displaying one fully developed leaf) for 19 days to achieve the optimum latency period [35]. Subsequently, each insect was placed on an individual uninfected wheat seedling for 24 h (inoculation period) and then fixed in absolute EtOH until use. Finally, after 25 days, the MRCV transmission to the wheat plants was individually determined by monitoring the presence of virus symptoms and by DAS-ELISA.

### RNA extraction and cDNA synthesis

Total RNA was extracted from each planthopper using the Trizol basic protocol (Invitrogen, CA, USA) with some modifications. First, previous to the extraction the sample was pulverized by freezing in liquid nitrogen and homogenized in a 1.5 ml microcentrifuge tube, and 800 μl of Trizol were added. Second, during the isopropanol precipitation 20 μg of glycogen (Sigma, USA) was added as a carrier. RNA concentration and purity were measured with a spectrophotometer (Thermo Scientific NanoDrop™ 1000, USA) and the integrity was checked by agarose gel electrophoresis. Finally, cDNA was synthesized using Superscript III and random primers from 500 ng of DNAse I (Invitrogen, CA, USA)-treated total RNA according to the manufacturer's instructions.

### Reference gene sequence isolation and primer design

For the isolation of *Delphacodes kuscheli *candidate gene sequences, degenerate oligonucleotides were designed from conserved regions of publicly available insect sequences of the actin (ACT), α-1-tubulin (TUB), elongation factor 1- α (EF1A), ribosomal protein S18 (RPS18), gliceraldehyde-3-phosphate dehydrogenase (GAPDH) and polyubiquitin C (UBI) genes. The designed oligonucleotides Ph.Act FW (5'- CACCAGGGWGTGATGGTSGGTATGG-3'), Ph.Act RV (5'- SACGTCGCACTTCATGATSGAGTTG-3'), Ph.Tub FW (5'- ACAACGARGCYATCTACGACATCTG-3'), Ph.Tub RV (5'- CTCCTTCCTCCATACCCTCWCCGAC-3'), Ph.eF1a FW (5'- GGAAATGGGYAARGGTTCCTTCAAG-3'), Ph.eF1a RV (5'- AGCCTGAGGGGCTTSTCAGTRGGTC-3'), Ph.RpS18 FW (5'- ARCGNAARGTBATGTTYGCCATGAC-3'), Ph.RpS18 RV (5'- TCTTNGABACACCAACAGTNCTTCC-3'), Ph.GAPDH FW (5'- TGGTNTACWTGTTCAARTAYGAYTC-3'), Ph.GAPDH RV (5'- ATTGGGNACTGGBACTCGGAARGCC-3'), Ph.UbiC FW (5'- GWGGTATGCARATCTTCGTSAARAC-3') and Ph.UbiC RV (5'- TGTAGTCRGARAGGGTTCTGCCATC-3') were used in Reverse Transcriptase-Polymerase Chain Reactions (RT-PCR) using *D. kuscheli *total RNA as template. The resulting amplified fragments were purified using QIAquick gel extraction kit (QIAGEN, Germany), cloned into pGemT Easy (Promega, USA) and sequenced. All *Delphacodes kuscheli *sequences were deposited in the GenBank database (Table 1). Finally, real time RT-qPCR specific oligonucleotides were designed from *D. kuscheli *sequences using Primer Express 3 software (Applied Biosystems, USA). The S18 rRNA primers were designed from the *Laodelphax striatellus *sequence [GenBank: AB085211]. The sequences of primers used for RT-qPCR analysis are listed on Table 1.

**Table 1:** *Delpacodes kuscheli *candidate genes for RT-qPCR

Name	Symbol	Function	GenBank Accession	Primer sequences (5' to 3')	A (bp)	E (%)
Actin	ACT	Cytoskeleton/Muscle contraction	HM565964	FW: AGCGTGGTTACAGCTTCACAAC RV: GGCCATTTCCTGTTCGAAGTC	101	76

α-1-tubulin	TUB	Cytoskeleton	HM565965	FW: GTTTTGAACCGGCCAATCAG RV: ACGTCTTTCGGCACGACATC	100	82

Elongation factor 1- α	EF1A	Translation	HM565967	FW: TTGAGGCTGGTATCTCGAAGAAC RV: GCTCGGTGGAGTCCATCTTG	111	79

Ribosomal protein S18	RPS18	40S ribosome subunit component	HM565969	FW: TGGCTCACCCAAGACAATACAAG RV: TTCAGCCTTTCCAGGTCATCAC	133	77

Gliceraldehyde-3-phosphate dehydrogenase	GAPDH	Glycolisys	HM565966	FW: CGCCAAGAAGGTGATCATTTC RV: CAGGAGGCGTTCGAGATGAC	115	65

Polyubiquitin C	UBI	Proteasome	HM565968	FW: CTGACCGGCAAGACGATTACG RV: GCCCTCCTTGTCCTGAATCTTC	84	82

18S rRNA	18S	40S ribosome subunit RNA component	ND	FW: TCGAAGGCGATCAGATACCG RV: TCTGGTTTCCCGGAAGCTG	105	55

List of *D. kuscheli *selected candidate genes names, assigned function, GenBank database accession codes, designed RT-qPCR primer sequences, length of the amplified fragment (A) and PCR efficiency as calculated by LinReg (E). ND: not determined.

### Quantitative real time PCR and data analysis

RT-qPCR experiments were carried out using the QuantiTec SYBR Green PCR kit (QIAGEN, Germany) according to the manufacturer's instructions, in an ABI7500 Real Time PCR System (Applied Biosystems, USA) with a standard program (1 min elongation time at 60°C). Reactions were performed in a 25 μl final volume reaction, using primers in a final concentration of 200 nM and 1 μl of a 1/10 dilution of the cDNA as template. No template was added to negative control reactions. Output results were exported as raw data, and introduced to the LinReg software [36] for baseline correction and PCR efficiency calculations. Resulting Ct values were used for expression stability analysis using the Microsoft Excel based tools geNorm 3.5 [37], Normfinder 0.953 [38] and Bestkeeper v1 [39] according to the developer's instructions. Statistical significance of Ct differences between treatments was calculated by the Mann-Whitney t test using the Graphpad Prism 5 software.

## Results and discussion

### Obtainment of MRCV-infective planthoppers and sample processing

The capacity of MRCV to replicate in plant phloem as well as in its planthopper vector tissues [35] suggests that the virus has the ability to regulate gene expression in both hosts. MRCV replication in plant phloem cells (where it produces severe cytopathic effects) and in planthopper vector tissues (where it establishes a persistent propagative infection) probably involves a differential regulation of gene expression in each case. To study these still unknown gene regulation processes in insects, reliable reference genes must be selected as internal controls to perform gene expression analysis. Consequently, planthoppers undoubtedly able to transmit MRCV are needed to analyze variations on potential reference gene expression levels with respect to naïve insects. One-to-one transmission assays were performed to obtain MRCV-infective insects as described in Methods. As a result, only 4 out of 50 independently evaluated insects were capable of successfully transmitting MRCV to wheat. This transmission efficiency was similar to the reported previously for this kind of assays [35]. Only planthoppers able to transmit MRCV to wheat plants (infective insects) were kept for subsequent RT-qPCR studies along with control naïve insects subjected to the same procedure but always fed on uninfected plants.

In parallel, four different methods for insect storage were tested: immediate freezing in liquid nitrogen followed by storage at -70°C or stored for 10 days in absolute ethanol, acetone or Trizol. Next, RNA extraction was performed from each planthopper using a Trizol protocol adapted to the small size of the samples (see Methods). After measuring the resulting RNA concentration and analyzing its integrity, ethanol storage was chosen for further experiments because it was a simpler procedure and gave rise to good RNA quality (Additional file 1).

### Isolation of partial sequences of candidate *Delphacodes kuscheli *genes for the design of qPCR primers

Seven housekeeping genes implicated in diverse cellular processes and commonly used as internal references for RT-qPCR in animals were selected as candidates for expression stability analysis. Partial sequences of *Delphacodes kuscheli *genes homologous to these candidate genes were obtained by RT-PCR with degenerated oligonucleotide primers (see Methods). The amplified actin (ACT), α-1-tubulin (TUB), elongation factor 1- α (EF1A), ribosomal protein S18 (RPS18), glyceraldehyde-3-phosphate dehydrogenase (GAPDH) and polyubiquitin C (UBI) *D. kuscheli *sequences showed 95%, 86%, 92%, 92%, 90% and 84% identity to *Nilaparvata lugens *ESTs sequences, respectively, and were deposited in the GenBank database (Table 1).

Next, RT-qPCR oligonucleotides were designed for each of the *D. kuscheli *isolated gene fragments. Owed to the high conservation of ribosomal RNA sequences, the 18S rRNA primers were based on an available *Laodelphax striatellus *sequence [GenBank: AB085211]. The designed RT-qPCR primers (Table 1) were able to efficiently amplify a single product of the expected size and did not show primer dimers formation in RT-PCR experiments (Additional file 2). In addition, a primer amplification performance test by real time RT-qPCR showed that all primer combinations yield a single amplicon and the absence of primer dimers formation in the dissociation step (data not shown). The PCR amplification efficiency of each set of primers was evaluated using the LinReg software which performs, for each individual sample, a baseline correction and a PCR efficiency calculation from the linear portion of the amplification curve slope by linear regression and then calculates a mean PCR efficiency for each primer pair [36]. The calculated PCR efficiency means ranged from 82% for UBI and TUB to 55% for 18S (Table 1). The 18S primer pair low PCR efficiency might have been caused by incomplete annealing due to mismatches, given that their design was based on a related species sequence.

### Expression stability analysis of the candidate genes and optimum number of genes for normalization

A real time RT-qPCR assay was carried out to examine the expression stability of the 7 candidate genes by the following procedure. Total RNA was extracted from the previously obtained MRCV-infective planthoppers along with control (naïve) individuals, and its concentration was precisely quantified with a spectrophotometer. As a mean for normalization of the sample input, equal amounts of total RNA (500 ng) from each planthopper were treated with DNAse I to remove possible genomic DNA contamination and then used for cDNA synthesis by retrotranscription as detailed previously. Parallel reactions without RT enzyme were performed and used for RT-PCR amplification as an additional control to check that no contaminating genomic DNA was present (Additional file 3). Finally, RT-qPCR experiments were carried as described in Methods. The full sample set including both infective and naïve planthoppers was subjected to each gene expression analysis to avoid between-run variations and three independent technical replicates were performed for each sample in all the experiments. The raw data obtained from each experiment was processed using the LinReg software for baseline correction and the resulting cycle threshold (Ct) values were used in candidate gene expression stability analysis by three different methods using geNorm [37], Normfinder [38] and Bestkeeper [40] tools.

**Figure 1:**
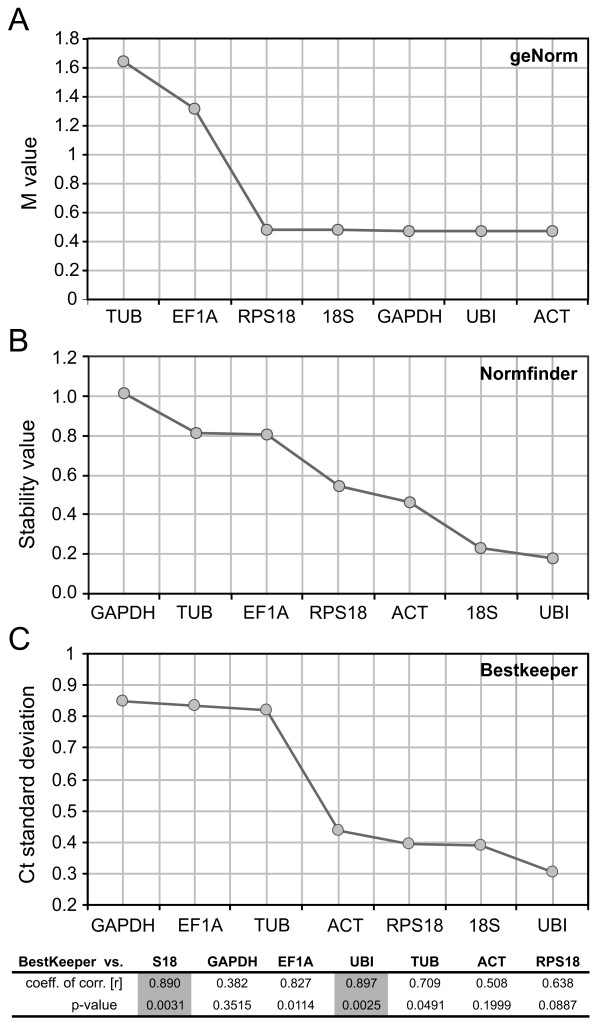
**Stability analysis of the candidate reference genes in *Delphacodes kuscheli *adult planthoppers**. The housekeeping genes were ranked according to their expression stability by (A) geNorm, (B) Normfinder and (C) BestKeeper statistical tools. In the three plots, genes were ordered from least (left) to most (right) stable. Pair-wise correlation analysis between the candidate reference genes and the calculated BestKeeper index is also shown (C, bottom) and the higher correlated genes were highlighted in grey.

**Figure 2:**
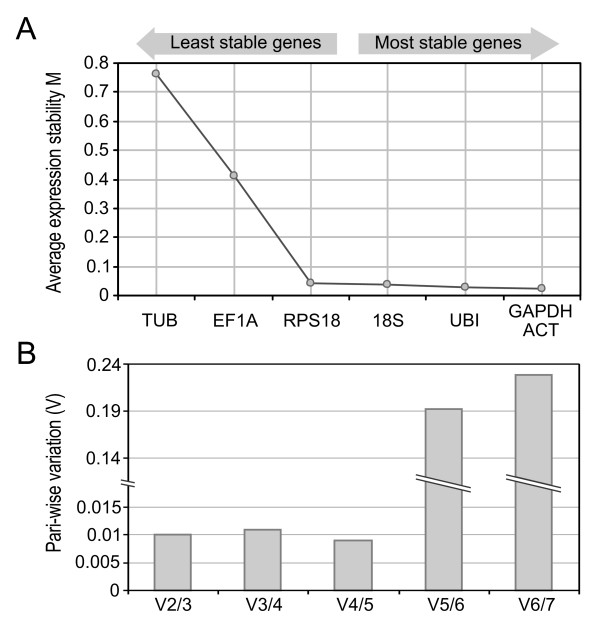
**Stepwise exclusion and pair-wise variation (v) analysis by GeNorm**. (A) Stepwise exclusion analysis in which the least stable (highest M value) gene is excluded sequentially (from left to right) until the most stable pair of genes remains. (B) Pair-wise variation analysis showing the normalization factor (NF) variability (V) that results from including an additional gene to the NF calculation. A V increase corresponds to a decrease in NF reliability.

GeNorm is a Visual Basic Application (VBA) for Microsoft Excel that ranks the genes under study by assigning a stability index M to each of them based on the average pair-wise variation of a gene compared to the rest of the studied genes [37]. According to the ranking of stability generated, ACT was the most stable gene with M= 0.4738, followed by UBI, GADPH, 18S and RPS18 with similar M coefficients. On the other hand, EF1A and TUB were listed as the most unstable genes with M > 1.2 (Figure 1A). Next, by performing a stepwise exclusion of the least stable gene and recalculating the M value for the remaining genes, geNorm detected that the most stable pair of reference genes was ACT/GAPDH (Figure 2A) which displayed the first and third place in the individual stability ranking. Finally, starting with the two most stable genes, the program calculates the pair-wise variation V of two consecutive normalization factors (NF) that result from stepwise introduction of another gene (the one with the lowest M remaining) [37]. This analysis showed that if we consider the generally adopted threshold of V = 0.15 [37,41,42], the inclusion of a third gene does not result in any appreciable improvement of the normalization factor. Moreover, the V values resulting from adding a third, fourth and fifth gene were low (V < 0.015) and the threshold was only reached when adding the two most unstable genes (Figure 2B). Thus, these results indicated that only the 2 most stable genes are needed for a reliable normalization.

The Normfinder VBA program analyzes gene expression stability by means of a mathematical model, focusing in the inter- and intra-group variation [38]. This approach ranked UBI as the most stable gene (stability value = 0.1754) followed by 18S, ACT and RPS18 (Figure 1B). This is in accordance to geNorm results, which situated UBI as the second most stable gene after ACT. However, Normfinder ranked GAPDH as the most unstable gene, whereas geNorm considered it the third most stable. This difference, due to the singular processing and assumptions of each statistical approach, indicates that it is strongly recommended to carry out more than one analysis when studying expression stability of putative reference genes.

Finally, BestKeeper Excel tool, analyzes each gene's expression variability by calculating the Ct set standard deviation (SD) and coefficient of variance (CV) and then by pair-wise comparison calculates the correlation between the genes and with the Bestkeeper index [40]. This application ranked UBI as the least variable gene with a Ct standard deviation (SD) of 0.31 (1.24 fold change) followed by 18S with SD = 0.39 (1.31 fold change) and indicated that TUB, EF1A and GAPDH were the most variable genes (Figure 1C). When pair-wise comparing the calculated BestKeeper index against each of the candidate genes, UBI and 18S also showed the higher correlation, with R > 0.89 and p = 0.003 (Figure 1C). It is worthy noticing that according to this analysis, none of the genes can be considered inconsistent since all of the SD values were lower than 1 [40].

BestKeeper and Normfinder yielded comparable results, selecting UBI and 18S as the two most stable genes and GAPDH as the least stable one (Figure 1B and 1C). On the other hand, the geNorm ranking was mostly dissimilar, positioning GAPDH as the third most stable gene (Figure 1A). Nevertheless, with the exception of this conflictive gene, all three approaches clearly separated the 7 candidate genes in two distinct groups according to their stability indexes: one group of a low variability and high stability composed of UBI, ACT, 18S and RPS18 and a second one more variable and unstable composed of EF1A and TUB (Figure 1). Taken the three method's output together, the results indicate that UBI is the most reliable reference gene for quantitative analysis of MRCV-infective planthopper mRNA levels by RT-qPCR. If two (or more) genes are used for normalization, 18S and ACT are the most suitable choices as a second reference. In agreement, the ACT gene has been recently employed as an internal control for the *Tomato spotted wilt virus *(TSWV) quantification in individual thrip vectors, although in this case the validation of its expression stability was not previously showed [43].

### Expression profiles of the candidate genes

**Figure 3:**
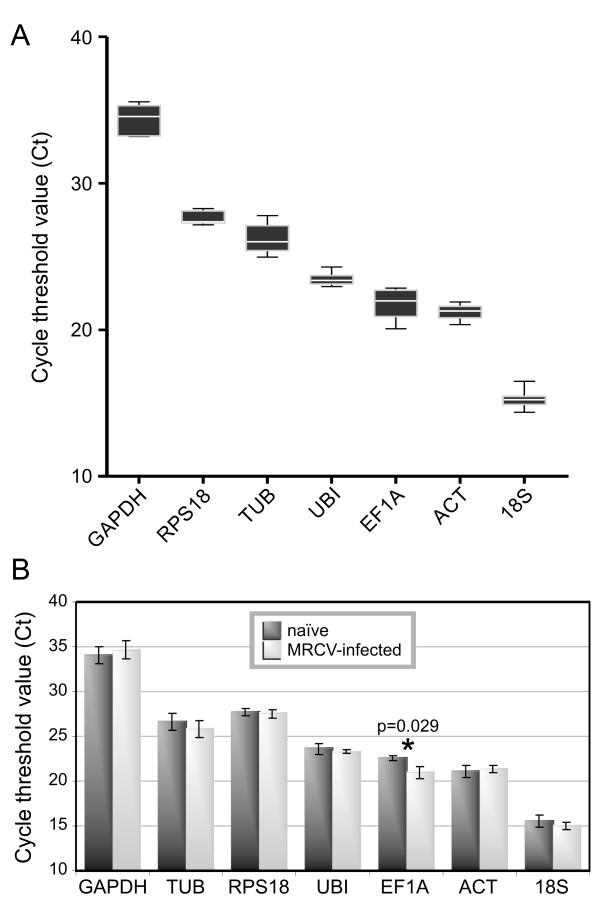
**Expression levels of the candidate reference genes**. (A) Expression levels displayed as cycle threshold (Ct) values of the genes used in this study, represented in a box and whisker diagram. Whiskers represent the maximum and minimum values. The genes were ordered from the least (higher Ct, to the left) to the most abundantly expressed (lower Ct, to the right). (B) Comparison of the expression levels of candidate reference genes in naïve versus MRCV-infective planthoppers. Differences between treatments were statistically evaluated for each gene by the Mann-Whitney t test.

Finally, the overall cycle threshold values (Ct) obtained for the different genes were compared. As shown in Figure 3A, the Ct values of the candidate genes were distributed from 15 to 34, in which the lowest Ct (and highest expression) corresponded to 18S and the highest Ct to GAPDH. The high expression level of the 18S rRNA is expected as it is one of the most abundant RNA species in the cell. Moreover, ACT second lowest Ct is in agreement with the usually abundant actin mRNA in cells [20]. It is important to note that the four genes consensually detected as the most reliable (UBI, 18S, ACT and RPS18) covered a relatively wide Ct range (15-28). It is convenient to count with several reference genes of diverse Ct values, because the use of internal controls with a Ct close to that of the gene whose expression is being studied is preferable.

Because the expression of genes classically considered as housekeeping has been found to be altered upon virus infection [22,44], we analyzed if MRCV infection altered the expression of any of the 7 candidate genes. The Ct values obtained for each gene were compared in MRCV-infective against naïve planthoppers and the statistical significance of Ct differences between treatments was calculated by the Mann-Whitney t test using the Graphpad Prism 5 software. Interestingly, EF1A expression was significantly higher (p < 0.05) in MRCV-infective planthoppers (Figure 3B). This fact explains the instability of EF1A gene expression previously detected when using a pool of infective and naïve insects (Figure 1). In agreement, EF1A gene expression has been reported to be 97.8 times more abundant in RSV-infected versus naïve small brown planthoppers [22]. It would be interesting to extend this study to a higher number of *Delphacodes kuscheli *genes, including antiviral genes of the innate immunity and RNAi pathways, to further establish if their expression is altered during persistent MRCV replication in its planthopper vector.

## Conclusions

Insect genomics is a growing area of research. In particular planthoppers transmit many important viral diseases and the molecular basis underling insect-virus interactions are relatively poorly studied. Here, a RT-qPCR platform useful for gene expression analysis in virus infected planthoppers was developed. The evaluation of several *D. kuscheli *housekeeping genes expression stability showed that the UBI gene is the most reliable as internal reference control for mRNA quantification in MRCV-infective planthoppers. On the other hand, the EF1A gene was upregulated in MRCV-infected planthoppers so it shouldn't be used as an internal control. Our work is the first report on reference gene selection in virus-infected insects, and might serve as a precedent for future gene expression studies on virus-planthopper pathosystems. Particularly, these results will surely contribute to the study of gene expression profiles in *D. kuscheli *in the context of MRCV infection. The RT-qPCR platform developed along this work will also be useful for normalizing viral titers in insect vectors, contributing to the already developed platforms for viral quantification in vectors and to new molecular studies of transmission and epidemiology [45-50]. Finally, the study of gene expression patterns in virus-transmitting arthropods will not only improve our knowledge of the virus-vector pathosystems but might also impact in the design of new strategies for virus control.

## List of abbreviations

RT-qPCR: real-time reverse transcription polymerase chain reaction.

## Competing interests

The authors declare that they have no competing interests.

## Authors' contributions

GAM performed the stability analysis and expression profiles of the candidate genes. MS maintained the MRCV inocula and performed one-to-one transmission assays to obtain MRCV-infective insects. VCM designed degenerated primers and amplified, cloned and analyzed the sequence of *D. kuscheli *reference genes. GAT supervised the virus transmission experiments and the insect rearing. MdV and GAM participated in the conception and design of the studies and wrote the manuscript. All authors read and approved the final manuscript.

## Supplementary Material

Additional file 1 **Analysis of different sample storage conditions**. Four storage conditions were assayed: three individual samples were frozen in liquid nitrogen and kept at -70°C or stored during 10 days in absolute ethanol, acetone or Trizol. Next total RNA was extracted and analyzed by agarose gel electrophoresis. Ribosomal RNA is indicated with an arrow. Click here for file

Additional file 2 **Analysis of the performance of the designed primers in regular RT-PCR reactions**. Amplification products of the different candidate genes in five samples (1-5) after regular RT-PCR using the specific primers listed on Table 1. (-) stands for control RT-PCR reactions where no cDNAs were added. DNA markers are indicated to the right. Click here for file

Additional file 3 **RT-PCR control reactions to assay for genomic DNA contamination on the *D. kuscheli *total RNA samples**. RT-PCR amplification of RPS18 performed in individual samples by using cDNA synthesized with or without the RT enzyme. A control RT-PCR reaction with no cDNA was carried out (-). Click here for file
