# Supplementary figures and images for: Reference gene selection for gene expression studies using RT-qPCR in virus-infected planthoppers
Source: Virol J. 2011 Jun 16;8:308. doi: 10.1186/1743-422X-8-308 (PMC3142240; doi:10.1186/1743-422X-8-308)

**-70°C**

**Ethanol**

**Acetone**

**Trizol**

1

2

3

1

2

3

1

2

3

1

2

3

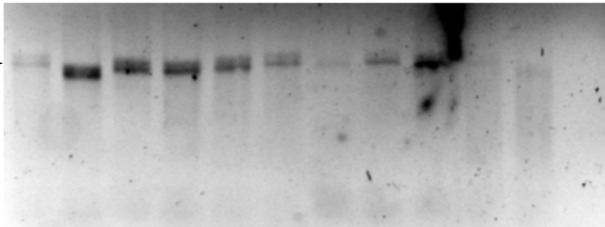

Supplement: Additional file 1 — Analysis of different sample storage conditions. Four storage conditions were assayed: three individual samples were frozen in liquid nitrogen and kept at -70°C or stored during 10 days in absolute ethanol, acetone or Trizol. Next total RNA was extracted and analyzed by agarose gel electrophoresis. Ribosomal RNA is indicated with an arrow. [file 1743-422X-8-308-S1.PDF]

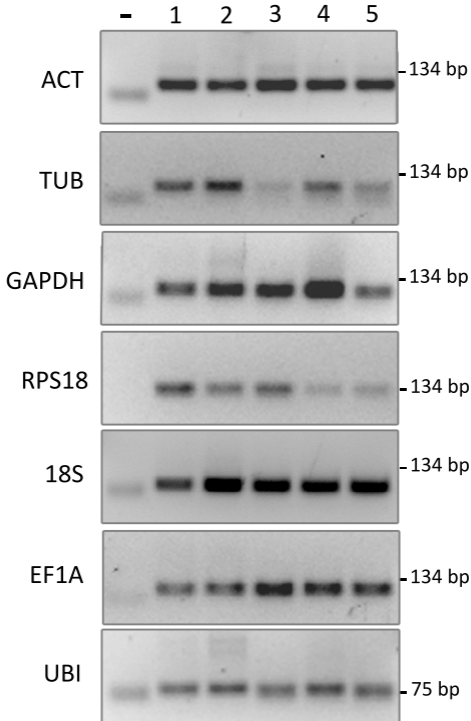

Supplement: Additional file 2 — Analysis of the performance of the designed primers in regular RT-PCR reactions. Amplification products of the different candidate genes in five samples (1-5) after regular RT-PCR using the specific primers listed on Table 1. (-) stands for control RT-PCR reactions where no cDNAs were added. DNA markers are indicated to the right. [file 1743-422X-8-308-S2.PDF]

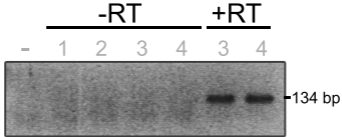

Supplement: Additional file 3 — RT-PCR control reactions to assay for genomic DNA contamination on the D. kuscheli total RNA samples. RT-PCR amplification of RPS18 performed in individual samples by using cDNA synthesized with or without the RT enzyme. A control RT-PCR reaction with no cDNA was carried out (-). [file 1743-422X-8-308-S3.PDF]
